# How resource abundance and resource stochasticity affect organisms’ range sizes

**DOI:** 10.1186/s40462-025-00546-5

**Published:** 2025-03-20

**Authors:** Stefano Mezzini, Christen H. Fleming, E. Patrícia Medici, Michael J. Noonan

**Affiliations:** 1 Okanagan Institute for Biodiversity, Resilience, and Ecosystem Services, The University of British Columbia Okanagan, Kelowna, BC Canada; 2 Department of Biology, The University of British Columbia Okanagan, Kelowna, BC Canada; 3 Department of Biology, University of Central Florida, Orlando, Florida 32816 USA; 4 Smithsonian Conservation Biology Institute, National Zoological Park, 1500 Remount Rd., Front Royal, VA 22630 USA; 5 Lowland Tapir Conservation Initiative (LTCI), Instituto de Pesquisas Ecológicas (IPÊ), Rodovia Dom Pedro I, km 47, Nazaré Paulista, São Paulo 12960-000 Brazil; 6 IUCN SSC Tapir Specialist Group (TSG), Campo Grande, Brazil; 7 Escola Superior de Conservação Ambiental E Sustentabilidade (ESCAS/IPÊ), Rodovia Dom Pedro I, km 47, Nazaré Paulista, São Paulo 12960-000 Brazil; 8 Department of Computer Science, Math, Physics, and Statistics, The University of British Columbia Okanagan, Kelowna, BC Canada

**Keywords:** Energetics, Resource abundance, Resource stochasticity, Environmental stochasticity, Home range, Range size, Movement behavior, ctmm

## Abstract

**Background:**

From megafauna to amoebas, the amount of space heterotrophic organisms use is thought to be tightly linked to the availability of resources within their habitats, such that organisms living in productive habitats generally require less space than those in resource-poor habitats. This hypothesis has widespread empirical support, but existing studies have focused primarily on responses to spatiotemporal changes in mean resources, while responses to unpredictable changes in resources (i.e., variance in resources or resource stochasticity) are still largely unknown. Since organisms adjust to variable environmental conditions, failing to consider the effects of resource unpredictability can result in an insufficient understanding of an organism’s range size.

**Methods:**

We leverage the available literature to provide a unifying framework and hypothesis for the effects of resource abundance and stochasticity on organisms’ range sizes. We then use simulated movement data to demonstrate how the combined effects of resource abundance and stochasticity interact to shape predictable patterns in range size. Finally, we test the hypothesis using real-world tracking data on a lowland tapir (*Tapirus terrestris*) from the Brazilian Cerrado.

**Results:**

Organisms’ range sizes decrease nonlinearly with resource abundance and increase nonlinearly with resource stochasticity, and the effects of resource stochasticity depend strongly on resource abundance. Additionally, the distribution and predictability of resources can exacerbate the effects of other drivers of movement, such as resource depletion, competition, and predation.

**Conclusions:**

Accounting for resource abundance and stochasticity is crucial for understanding the movement behavior of free-ranging organisms. Failing to account for resource stochasticity can lead to an incomplete and incorrect understanding of how and why organisms move, particularly during periods of rapid change.

**Supplementary Information:**

The online version contains supplementary material available at 10.1186/s40462-025-00546-5.

## Background

The amount of resources an organism is able to access is a strong determinant of its fitness. Resource limitations can cause individuals to experience a negative energetic balance, which can then result in lower fitness [1, 2], altered physiology [2–5], lower chance of reproduction [2, 6–8], and even death [9, 10]. Thus, many organisms adapt their behaviors and/or physiology in response to changes in local resource abundance to ensure their needs are met (e.g., soil amoebae *Dictyostelium* spp.: [11], plants: [12], and animals: [13]).

While there are many ways that individuals can respond to resource availability, movement represents one of the most readily available traits that motile species can adjust [14, 16, 25]. The relationship between organisms’ movement and resource abundance has long been of interest to biologists. In his seminal paper, Burt [17] considered the search for food as the primary driver for movement within an organism’s home range. Three decades after, Southwood [18] suggested that change in resource abundance drives how organisms decide where to live and when to reproduce. Two years later, Harestad and Bunnel [13] proposed that the simplest relationship between resource abundance and an organism’s home-range size is

1 $$\begin{aligned} H = C / R, \end{aligned}$$

where $H$ is the organism’s home-range size, $C$ is the organism’s resource consumption rate (kcal day$^{-1}$), and $R$ is the resources the organism can access (kcal day$^{-1}$ unit area$^{-1}$). Harestad and Bunnel’s model is simple to conceptualize, and it allows for testable predictions, but few studies are structured around a set of theoretical expectations such as Harestad and Bunnel’s hypothesis. Many researchers have since demonstrated that organisms adapt their range sizes in response to resource abundance, but results are typically reported as independent, novel findings. Perhaps more problematic is the fact that, while much work has been done on estimating organisms’ responses to changes in mean resource abundance, there is little information on how organisms respond to unpredictable changes in resources (i.e., resource stochasticity, but see: [19–22]). Thus, there remains a need for a clear, unifying hypothesis of the effects of both resource abundance and stochasticity on organisms’ range sizes.

Here, we refer to a location’s average amount of resources as “resource abundance”, while we use the phrase “resource stochasticity” to indicate the variability in resources after accounting for changes in the mean. We argue that, on its own, a habitat’s resource abundance is not sufficient to assess the habitat’s quality, nor make predictions about how much space an organism might use. To see this, consider, for instance, a herbivore grazing in a grassland with relatively low but constant forage availability (i.e., low mean and variance). The animal may require a large but constant home range size as it moves between patches in search of food. If, instead, it lived in a desert with equally scarce forage but rare, sudden, and strong pulses of resources (i.e., low long-term mean and high stochasticity), it may switch between dispersal in search for high-resource patches and short-term range residency within patches (*sensu* [15], see [23–25]). Previous studies suggest that resource stochasticity may decrease organisms’ fitness and landscapes’ energetic balances (e.g., [26]), but there is still limited empirical evidence to support this hypothesis (but see: [21, 27, 28]).

In this paper, we illustrate how an organism’s range size can be expected to depend on both the abundance and unpredictability of resources. First, we set the theoretical background necessary for the successive sections by introducing key concepts and notation. Next, we provide a review of the effects of resource abundance on range sizes while suggesting a simple and unifying hypothesis. Afterwards, we provide a review of the effects of resource stochasticity on organisms’ range sizes while suggesting a second simple and unifying hypothesis. Subsequently, we support the hypothesis using quantitative, simulated responses in range size to changes in resource abundance and stochasticity. Finally, we demonstrate how this framework can be used in practice to describe the movement ecology of a lowland tapir (*Tapirus terrestris*) from the Brazilian Cerrado [29].

## Resources as a random variable

Resources (e.g., food, water, shelter, heat) are often unpredictable (and difficult to quantify), since they depend on various factors which cannot be accounted for easily, including climate [7, 30, 31], weather [31, 32], competitive pressure [33, 34], and differences in energetics at among individuals [7] and species [35]. Thus, it is possible to treat the amount of resources $R$ at a given point in time ($t$) and space (location vector $\vec u$) as a random variable, denoted as $R(t, \vec u)$. Treating resources as a random variable allows us to leverage techniques from probability theory and statistics, such as the expectation of a random variable (i.e., its mean) and its variance around the mean. We indicate the expected value and variance of random variable $R$ using $\text {E}(R)$ and $\text {Var}(R)$, respectively, and we use $\mu (t, \vec u)$ and $\sigma ^2(t, \vec u)$ to indicate them as functions of time ($t$) and space ($\vec u$). Appendix A defines and expands on the concepts of probability distributions, expected value, variance, and provides examples of them for Gamma and Beta distributions.

### Effects of resource abundance, $\text {E}(R)$

While organisms’ needs vary greatly between taxonomic groups, some needs are essential for the growth, survival, and reproduction of most organisms. All heterotrophic organisms require sources of chemical energy (i.e., food), water, and various limiting nutrients [36–38]. As the abundance of essential resources fluctuates, motile organisms can move to new locations or ‘patches’ to meet their requirements [15, 39], but movement also increases energetic needs [40].

**Fig. 1:**
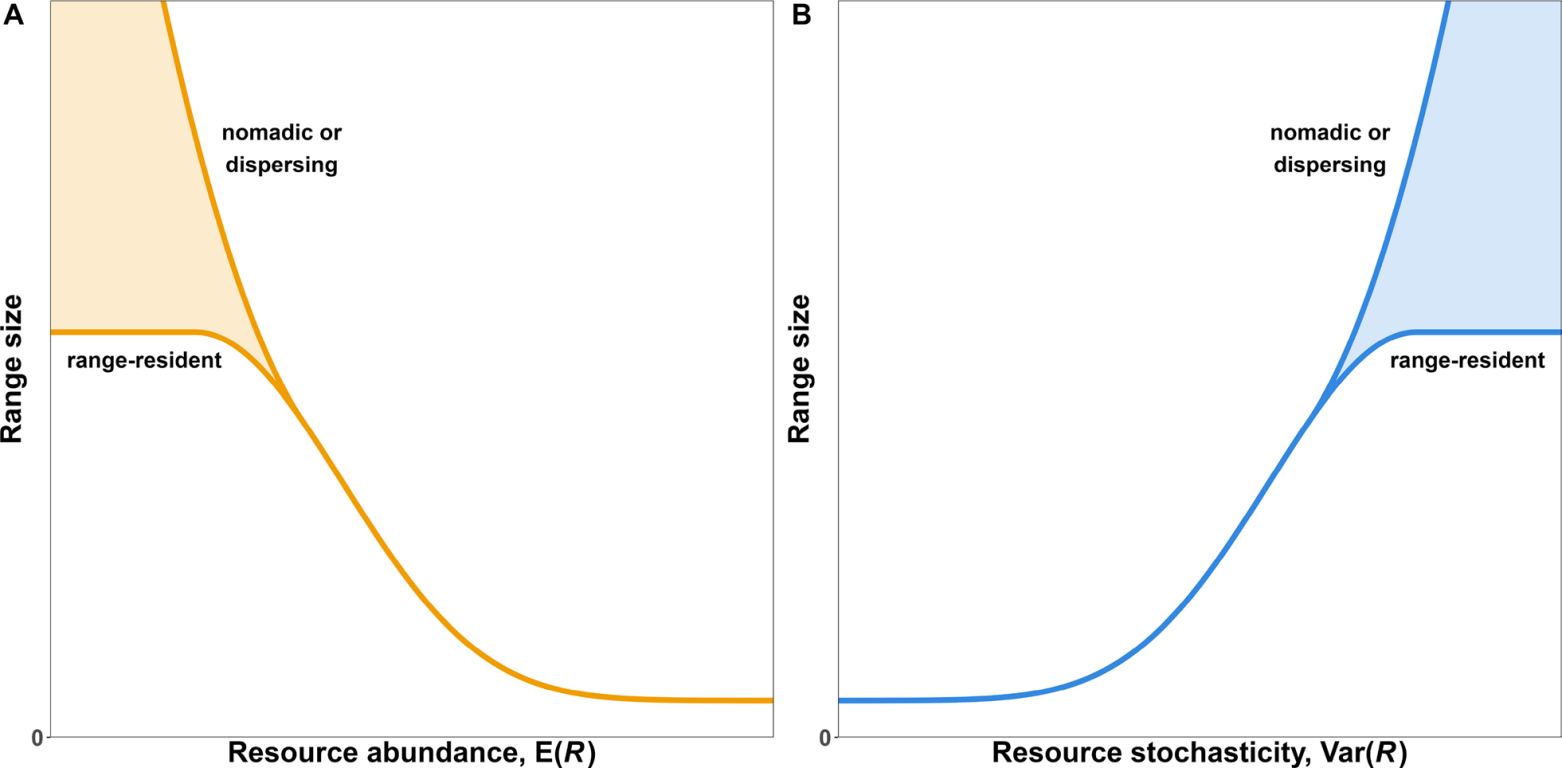
Hypothesized range size of an organism as a function of **A** resource abundance and **B** resource stochasticity. We expect low values of $\text {E}(R)$ and large values of $\text {Var}(R)$ to result in a large range, since organisms are forced to explore large areas to collect the resources they require to survive, whether they be range-resident, nomadic, or migratory. As $\text {E}(R)$ increases or $\text {Var}(R)$ decreases, range size should decrease nonlinearly until it reaches the minimum amount of space required by the organism to survive. Note that the relationship between range size and both $\text {E}(R)$ and $\text {Var}(R)$ cannot be of the form $H = \beta _0 + \beta _1 \text {E}(R) + \beta _2 \text {Var}(R)$ because it would require range size to be negative for high values of $\text {E}(R)$ or low values of $\text {Var}(R)$

When $\text {E}(R)$ is high, we expect organisms’ ranges to be relatively small and near the smallest amount of space required to survive (see Fig. 1A as well as: [27, 28, 41]). Like Harestad and Bunnel [13], we also expect organisms’ range sizes to increase nonlinearly as $\text {E}(R)$ decreases, but we highlight that organisms may adopt different behaviors at low values of $\text {E}(R)$. These behaviors include maximal home range expansion (home range size is limited by vagility, habitat structure, competition, and predation, e.g., [33, 34, 42, 43]), migration [44–46], and nomadism [23, 25, 47, 48]. It is unclear when organisms switch from range residency to migration or nomadism (or vice-versa), but understanding the gradient among these types of movement is necessary for quantifying the effect of resource abundance on organisms’ range size and movement behavior (mammals: [49], moose, *Alces alces*: [23], eagles, *Haliaeetus leucocephalus*: [24, 50], lesser flamingos, *Phoeniconaias minor*: [51]).

Overall, the hypothesis that range size decreases with resource abundance, $\text {E}(R)$, is commonly accepted and well supported, but many studies assume a linear relationship (e.g., [21, 41, 52–54]). This is problematic because, conceptually, the relationship between range size and $\text {E}(R)$ must be nonlinear, since: (1) there is an upper limit to how much space an organism is able to explore in its finite lifetime and (2) the minimum amount of space it requires to survive is necessarily greater than zero (see [27, 28, 55–57], and contrast them to the earlier references that assume a linear relationship between $H$ and $R$). Consequently, we suggest analysts use models that account for this nonlinearity when estimating the effects of resource abundance on range size. While the relationship may be approximately linear for some range of $\text {E}(R)$, this assumption often does not hold for low or high values of $\text {E}(R)$ (e.g., [52]). Additionally, identifying inflection points in nonlinear relationships can help understand the pressures and limitations of increasing range size.

### Effects of resource stochasticity, $\text {Var}(R)$

Assuming resource stochasticity is constant over time and space can be a useful simplification of relatively stable environments or when information on how $\text {E}(R)$ changes is limited and estimating changes in $\text {Var}(R)$ is unreasonable. However, such an assumption is likely not realistic, since $\text {Var}(R)$ often differ across space and over time. Generally, bounded quantities have correlated means and variances, as in the case of random variables that are strictly positive (e.g., Gamma and Poisson) or fully bounded (e.g., Beta). For example, prey abundance in a given area over time may approximately follow a Poisson distribution, which implies that the mean and variance will be approximately equal. When prey are scarce, the variance will also be low, and when prey are abundant the variance will also be high. This occurs because the behavior, fitness, and predator–prey dynamics of many prey are more stochastic than those of few prey [58]. Similarly, in the case of fully bounded random variables, the variance is generally lowest when the mean is near either boundary. For example, successful predation events are predictably scarce if the probability of capture is near 0, predictably common if the probability is near 1, and most stochastic if the probability is near 0.5 (i.e., as far as possible from both 0 and 1; see [59]). See Appendix A for more information.

Recognizing changes in $\text {Var}(R)$ helps account for the residual, fine-scale variation in $R$ after accounting for trends in the large-scale average $R$ (e.g., variations in plant phenology between years after accounting for mean seasonal trends, see [60]). However, when both $\text {E}(R)$ and $\text {Var}(R)$ change over time (fig. A2), disentangling changes in $\text {E}(R)$ and $\text {Var}(R)$ is not simple [61]. Statistically, this confound occurs because the more change one attributes to $\mu (t, \vec u)$ (i.e., the wigglier it is), the smaller $\sigma ^2(t, \vec u)$ becomes. Conversely, the smoother $\mu (t, \vec u)$ is, the larger $\sigma ^2(t, \vec u)$ becomes. Biologically, it is important because an organism’s perception scale determines whether it attributes a change in $R$ to a trend in $\text {E}(R)$ or as a stochastic event (i.e., due to $\text {Var}(R)$; see [60]). An organism’s perception of changes in $R$ will also depend strongly on the its cognitive capacities and memory [9, 62–65]. Whether an organism is able to predict trends in $\sigma ^2(t, \vec u)$ or not, environmental variability is thought to reduce a landscape’s energetic balance [26], which, in turn, decreases organisms’ fitness (e.g., [10]) and increases their range size. While this behavioral response occurs with both predictable and unpredictable stochasticity, extreme and rare events are more likely to have a stronger effect due to their unpredictability and magnitude [66, 67]. A few recent studies support these hypotheses [22, 26, 31, 48, 68], but many of them are limited in geographic and taxonomic scales or fail to account for nonlinear relationships, so the extent to which these preliminary findings can be generalized is currently unknown. Thus, there remains a need for developing a more complete understanding of how organisms’ range sizes changes with environmental stochasticity.

Similarly to $\text {E}(R)$, we hypothesize $\text {Var}(R)$ has a nonlinear effect on an organism’s range size. When $\text {Var}(R)$ is low enough that $R$ is relatively predictable, we expect organisms to be range-resident with small home ranges, and we do not expect small changes in $\text {Var}(R)$ to have a noticeable effect. As resources become increasingly unpredictable, we expect home range size to increase progressively faster (Fig. 1B) because: (1) as $\text {Var}(R)$ increases, the chances of finding low $R$ increase superlinearly, (2) the added movement required to search for food increases organisms’ energetic requirements, and (3) stochasticity reduces an organism’s ability to specialize and reduce competition for $R$ [69]. If resources remain highly unpredictable over long periods of time (e.g., multiple lifespans), organisms may evolve or develop new and consistent behaviors (e.g, nomadism) or adaptations (e.g., increased fat storage or food caching) to buffer themselves against times of unpredictably low $R$. Conversely, if changes in $\sigma ^2(t, \vec u)$ are sufficiently predictable, organisms may learn to anticipate and prepare for times of greater stochasticity by pre-preemptively caching food, reducing energetic needs, migrating, or relying on alternative food sources (e.g., [70]).

### Interactive effects of $\text {E}(R)$ and $\text {Var}(R)$

We have provided the case for why both $\text {E}(R)$ and $\text {Var}(R)$ should be expected to affect organisms’ range size, but we presented the two parameters as independent drivers of movement. However, organisms may respond to changes in $\sigma ^2(t, \vec u)$ more when resources are scarce than when they are abundant. Consequently, an organism’s movement behavior is likely to be a function of not only the marginal effects of $\text {E}(R)$ and $\text {Var}(R)$ but also their interactive effects. A highly unpredictable habitat may be very inhospitable if resources are poor, but $\text {Var}(R)$ may have little effect if resources are stochastic but always abundant. Thus, we expect $\text {Var}(R)$ to have a stronger effect on range size when $\text {E}(R)$ is low, and less of an effect when $\text {E}(R)$ is high. We explore this interaction effect more in the following section.

### Simulating responses to $\text {E}(R)$ and $\text {Var}(R)$

To evaluate our hypothesis of how organisms’ range sizes are affected by $\text {E}(R)$, $\text {Var}(R)$, and the interaction effect of $\text {E}(R)$ and $\text {Var}(R)$, we present the results from a series of quantitative simulations. To start, we used the ctmm package [71] for R [72] to generate 200 tracks (see Appendix B for sensitivity analyses) from an Integrated Ornstein-Uhlenbeck movement model (IOU model, see [73]). The IOU model’s correlated velocity produced tracks with directional persistence, but, unlike Ornstein-Uhlenbeck (OU) and Ornstein-Uhlenbeck Foraging (OUF) models, IOU models do not produce spatially stationary movement, so the organism is not range-resident. Consequently, each track is spatially unrestricted and can be interpreted as purely exploratory or memoryless movement.

Each of the 200 tracks were placed on a grid with common starting point $\langle 0, 0\rangle$ (fig. B1). Each time the simulated individual moved to a new cell, it collected $R$ resources sampled from a Gamma distribution. The mean and variance of the distribution were defined by a series of deterministic functions $\mu (t)$ and $\sigma ^2(t)$ (orange and blue lines in Fig. 3). The value of $t$ was constant within each set of 200 tracks, so the distribution $R$ was sampled from was independent of both the organism’s location and its time spent moving. Tracks were truncated once the organism reached satiety, and the organism was given enough time to return to $\langle 0, 0\rangle$ independently from the following track (section 2.1 of Appendix B). Finally, we fit an OUF movement model [74] to the set of tracks to calculate the 95% Gaussian home-range size using the formula

$$\begin{aligned}\hat{H}_{95\%} = -2 \log (1 - 0.95) \pi \hat{\varsigma }^2,\end{aligned}$$

where ${\hat{\varsigma }}^2$ is the positional variance estimated by the movement model.

We designed the simulations to estimate the effects of $\text {E}(R)$ and $\text {Var}(R)$ in simplistic environments where organisms could only respond by searching for longer periods of time. Consequently, we made the following assumptions:

1. Environments are homogeneous for a given $t$. Given $t$, $\text {E}(R) = \mu (t)$ and $\text {Var}(R) = \sigma ^2(t)$ are constant over space and within each set of 200 tracks, but $R$ is random and follows a $\text {Gamma}(\mu (t), \sigma ^2(t))$ distribution.
2. The are no external pressures on the simulated organism. Resources do not deplete, and there is no competition nor predator avoidance.
3. The organism has a fixed daily energetic requirement that is independent of movement rates, and it cannot alter its metabolism or physiology. Additionally, the organism does not have energetic reserves, so excess resources cannot be carried over to the next track or $t$.
4. The organism is range-resident and can only respond to changes in $\text {E}(R)$ and $\text {Var}(R)$ by altering its home-range size. The organism does not disperse or abandon a range.
5. The organism’s movement is simplistic. The organism’s movement speed and direction are stochastic and independent of $\text {E}(R)$ and $\text {Var}(R)$.
6. The organism has no perceptive range or memory. It is unable to detect, learn, or predict where resources are abundant (high $\text {E}(R)$) or reliable (low $\text {Var}(R)$) over time or space.
7. Animals only move to search for food or return to the center of their home-range after reaching satiety.

**Fig. 2:**
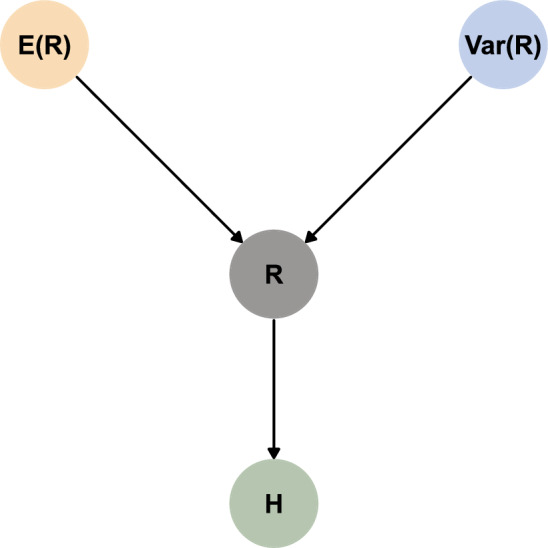
Directed acyclical graph assumed for inferring the causal effects of $\text {E}(R)$ and $\text {Var}(R)$ on the distributions of *R* and *H* in the simulations

Based on the assumptions above, we constructed the following causal model for the simulated effects of $\text {E}(R)$ and $\text {Var}(R)$ on $H$ (see Fig. 2 and [75]): $\text {E}(R)$ and $\text {Var}(R)$ were determined independently of each other, but they jointly determined the distribution of $R$, which, in turn, determined the distribution of $H$. Additional information is provided in Appendix B.

**Fig. 3:**
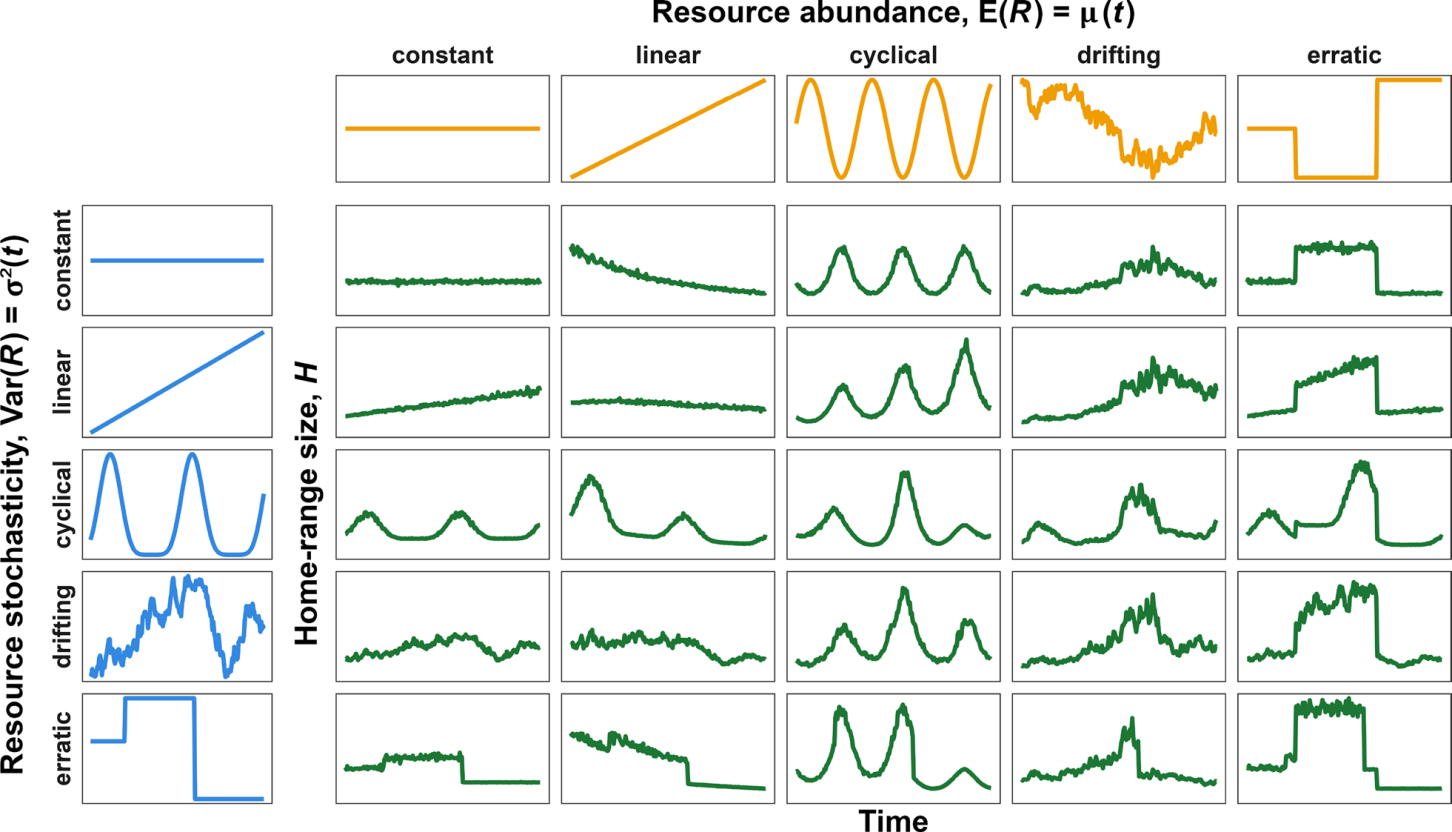
Simulated home-range sizes, *H*, of an organism living in habitats where the mean and variance in resources are constant, linearly increasing, cyclical, drifting, or erratic over time (but homogeneous over space for a given *t*). Note how *H* decreases nonlinearly as $\mu (t)$ increases and increases nonlinearly as $\sigma ^2(t)$ increases. Additionally, the variance in *H* is higher when $\mu (t)$ is lower or $\sigma ^2(t)$ is higher, and changes in $\sigma ^2(t)$ have greater impacts when $\mu (t)$ is low

Figure 3 shows how simulated home-range size, $H$, responded to changes in $\mu (t)$ and $\sigma ^2(t)$ in scenarios where both functions can remain constant, increase linearly, oscillate cyclically, drift stochastically, or change erratically. The top row (constant $\text {Var}(R)$) shows how $H$ varies for different trends in $\mu (t)$ while $\text {Var}(R)$ remains constant (like in fig. A1). As $\text {E}(R)$ increases at a constant slope (linear $\mu (t)$), $H$ decreases nonlinearly, with larger changes when $\text {E}(R)$ is low, until it approaches the minimum size required by the organism. Also note how the noise in the green lines also decreases as $\text {E}(R)$ increases.

The leftmost column of Fig. 3 (constant $\text {E}(R)$) illustrates the effects of $\text {Var}(R)$ on $H$ while $\text {E}(R)$ remains constant. Overall, both mean $H$ and the variance around it increase with $\sigma ^2(t)$ (most visible with constant $\text {E}(R)$ and linear $\text {Var}(R)$). Similarly to resource-poor periods, times of greater stochasticity require the organism to move over larger areas for longer periods of time. Additionally, the greater in uncertainty in how much time and space the organism will require to reach satiety, or indeed whether an organism living in highly stochastic environments can even reach satiety within a finite amount of time.

The remaining panels in Fig. 3 illustrate how $\text {E}(R)$ and $\text {Var}(R)$ jointly affect $H$ and how unintuitive the effects can be. Since $\text {E}(R)$ and $\text {Var}(R)$ have opposite effects on $H$, disentangling the effects can be particularly difficult when both parameters change in a correlated manner (e.g., linear $\text {E}(R)$ and $\text {Var}(R)$). When both $\text {E}(R)$ and $\text {Var}(R)$ increase linearly, $H$ initially increases since the effect of $\text {Var}(R)$ is stronger, but then decreases as the effect of $\text {E}(R)$ begins to dominate. Difficulties in disentangling the two effects are explored in greater depth in the case study in the following section.

**Fig. 4:**
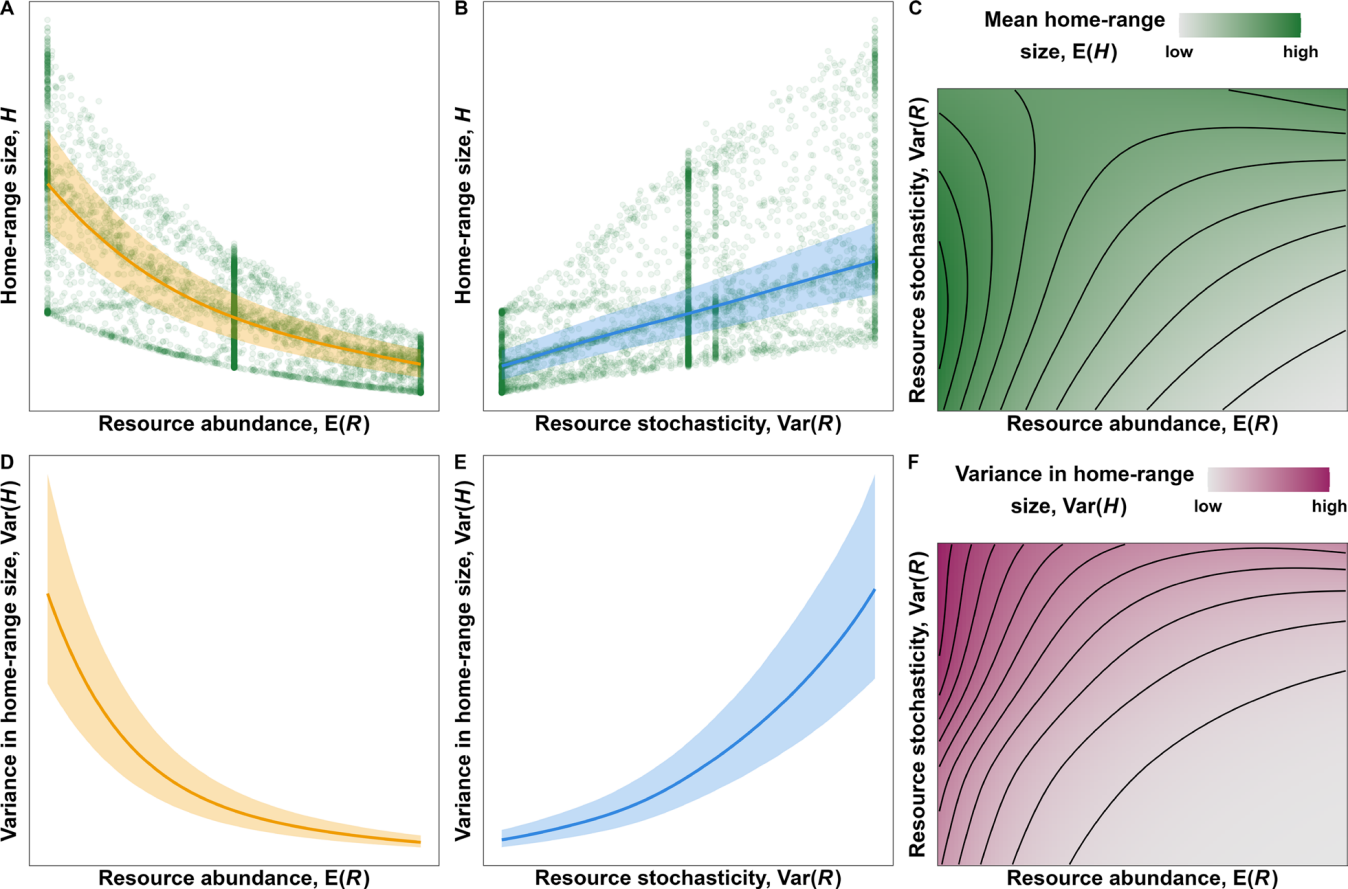
Effects of $\text {E}(R)$ and $\text {Var}(R)$ on on the mean (**A**–**C**) and variance (**D**–**F**) in simulated home-range size with 95% Bayesian credible intervals. While the estimated marginal effect of $\text {Var}(R)$ on $\text {E}(H)$ is sublinear (**B**), the effect of $\text {Var}(R)$ is superlinear for high values of $\text {E}(R)$ (**C**). The relationships were estimated using a Generalized Additive Model for Location and Scale with a Gamma location-scale family of distributions ($\mathtt {mgcv\!::\!gammals}$). Credible intervals were calculated using 10,000 samples from the posterior distribution while assuming multivariate Gaussian coefficients. Additional details on the model structure are provided in Appendix B

Although the temporal trends in Fig. 3 are complex and the effects of $\text {E}(R)$ and $\text {Var}(R)$ can be hard to disentangle, two simple relationships emerge when $H$ is shown as a function of either $\text {E}(R)$ or $\text {Var}(R)$, rather than time: $H$ decreases nonlinearly with $\text {E}(R)$ and increases with $\text {Var}(R)$ (panels A and B of Fig. 4). The estimated relationships thus follow the hypothesis we presented in Fig. 1, although we found that the effect of $\text {Var}(R)$ at average $\text {E}(R)$ was linear with a slight sublinear saturation at high values of $\text {Var}(R)$. However, notice that the effect of $\text {Var}(R)$ on $E(H)$ depends strongly on $\text {E}(R)$ (panel C): When $\text {E}(R)$ is low, $\text {E}(H)$ is high and $\text {Var}(R)$ does not have a strong effect, but when $\text {E}(R)$ is high the effect of $\text {Var}(R)$ on $\text {E}(H)$ is exponential. Similarly, $\text {E}(H)$ decreases exponentially with $\text {E}(R)$ except when $\text {Var}(R)$ is very high.

As expected by the changes in the spread of the points in panels A and B of Fig. 4, the variance in $H$, $\text {Var}(H)$, also depends on $\text {E}(R)$ and $\text {Var}(R)$ (Fig. 4D–F). Since we modeled $H$ using a Gamma family of distributions, we expected $\text {Var}(H)$ to increase with $\text {E}(H)$, but the location-scale model removes the assumption of a constant mean-variance relationship (i.e., constant coefficient of variation, $\frac{\mu (t)}{\sigma ^2(t)}$. This allowed us to show that the effect of $R$ on $\text {Var}(H)$ is much stronger than the effect of $R$ on $\text {E}(H)$. Consequences of these effects are explored in the discussion section.

## A case study on a lowland tapir in the Brazilian Cerrado

The simulations in the section above support the hypothesis we presented in the background section, but they are based on assumptions that are often not met in real natural environments. Organisms live in spatiotemporally heterogeneous and dynamic environments that promote the use of perceptual ranges, navigation, and memory. Together, these abilities result in selective space use that depends on resource availability [14] and resource depletion [15].

In this section, we test the hypothesis using empirical tracking data on a lowland tapir from the Brazilian Cerrado along with empirical estimates of $\text {E}(R)$ and $\text {Var}(R)$. We measure $R$ using Normalized Difference Vegetation Index [NDVI, see 76], a remote-sensed measure of landscape greenness, as a proxy for forage abundance. Appendix C contains additional information on how we modeled NDVI and the tapir’s movement using continuous-time movement models [71, 77] and autocorrelated kernel density estimation [78–80].

**Fig. 5:**
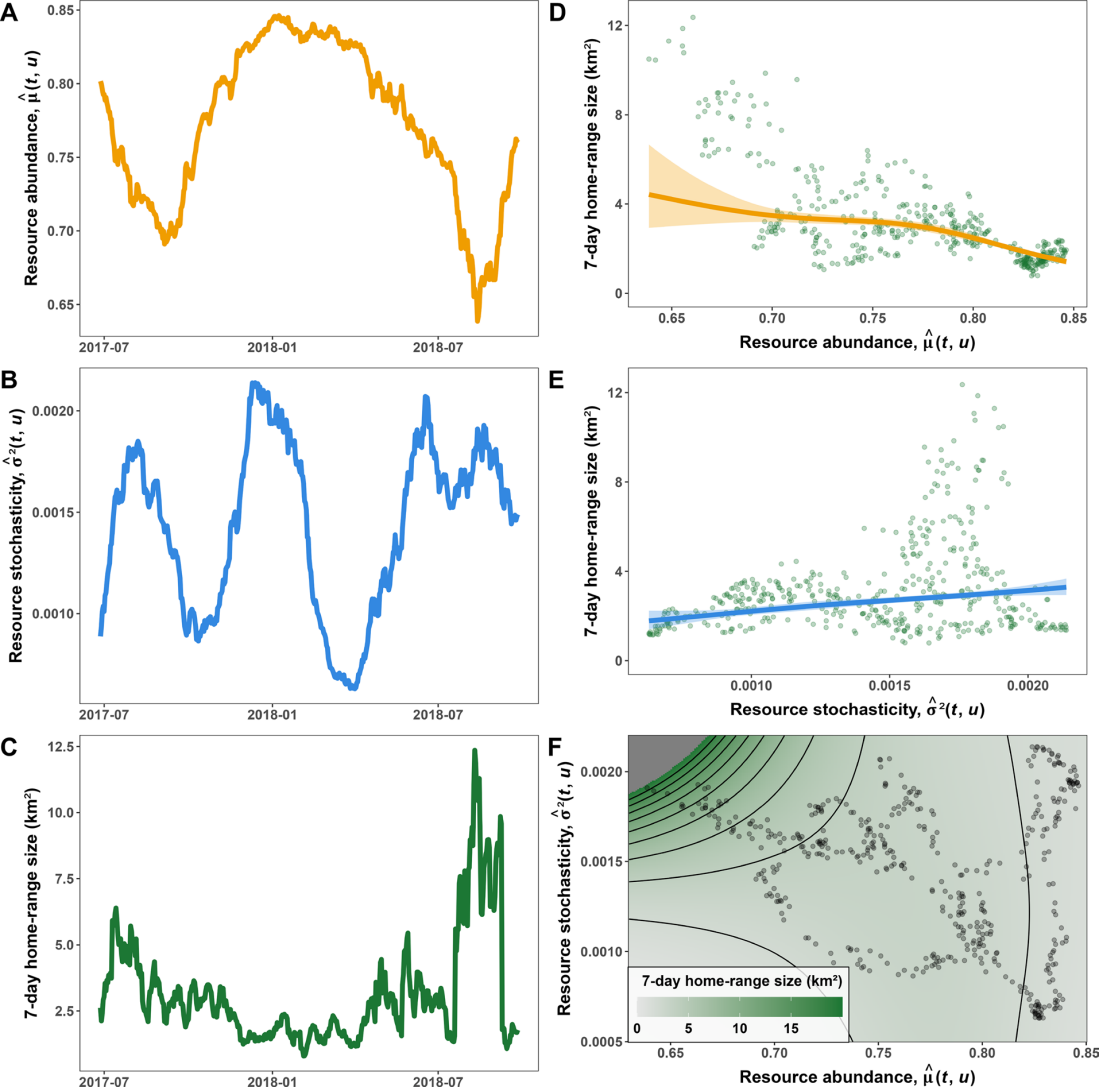
Effects of estimated $\mu (t, \vec u)$ and $\sigma ^2(t, \vec u)$ on the home-range size of a lowland tapir (*Tapirus terrestris*). **A** Trends in resource abundance over time, ${\hat{\mu }}(t, \vec u)$, estimated as the average mean NDVI at the locations visited by the tapir during a 7-day period. **B** Variance in resources over time, ${\hat{\sigma }}^{2}(t, \vec u)$, estimated as the average variance in NDVI at the locations visited by the tapir during a 7-day period. **C** Seven-day 95% home range estimated using Autocorrelated Kernel Density Estimation. **D**, **E** Estimated marginal effects of ${\hat{\mu }}(t, \vec u)$ and ${\hat{\sigma }}^{2}(t, \vec u)$ on home-range size. The model accounted for the marginal effects of ${\hat{\mu }}(t, \vec u)$, ${\hat{\sigma }}^{2}(t, \vec u)$, and their interaction effect. **F** Estimated home-range size in response to changes in both ${\hat{\mu }}(t, \vec u)$ and ${\hat{\sigma }}^{2}(t, \vec u)$. Note how the effect of ${\hat{\sigma }}^{2}(t, \vec u)$ is more pronounced when ${\hat{\mu }}(t, \vec u)$ is low. See Appendix C for additional information. The tapir movement data corresponds to the individual named “Anna" from the Cerrado sample of Medici et al. [29]

Figure 5 illustrates how a tapir in the Brazilian Cerrado adapted its 7-day home-range size to spatiotemporal changes in estimated $\mu (t, \vec u)$ and $\sigma ^2(t, \vec u)$ (telemetry data from the individual labelled as “Anna” in the dataset from [29]). Panels A and B show the changes in seven-day average mean and variance in NDVI, respectively, experienced by the tapir during the tracking period. The mean and variance in NDVI were estimated using a Generalized Additive Model for Location and Scale ([81]) with a Beta family of distributions (NDVI values ranged from 0.3534 to 0.9475). Panel C shows the changes in the tapir’s 7-day home range over time. All 457 of the 7-day windows had a minimum effective sample size of 7 range crossings (range: 7.7–69.6, see [82]), and 92% had resolvable (i.e., non-NA) home range crossing times, all of which were below 17 h. Note how the tapir uses more space during periods of lower NDVI (e.g., August 2017) and less space during periods with high NDVI (January 2018). Additionally, when resources are scarce and highly unpredictable (August 2018), the tapir uses up to 5 times more space than when resources are abundant and predictable (e.g., January 2018). Finally, panels D and E show the estimated (marginal) effects of ${\hat{\mu }}(t, \vec u)$ and ${\hat{\sigma }}^{2}(t, \vec u)$ on the tapir’s 7-day home-range size. Since ${\hat{\mu }}(t, \vec u)$ and ${\hat{\sigma }}^{2}(t, \vec u)$ are correlated (panel F) and spatiotemporally autocorrelated (panels A, B, and F), the effects of $R$ on $H$ should be modeled carefully. To avoid over-fitting the model, we constrained the smooth effects of ${\hat{\mu }}(t, \vec u)$ and ${\hat{\sigma }}^{2}(t, \vec u)$ and their interaction effect to a small basis size ($\mathtt {k = 3}$). Additional information is provided in appendix C. The results presented in panels D–F of Fig. 5 match our findings from the simulations (Fig. 4A–C): The tapir’s 7-day home range decreases with ${\hat{\mu }}(t, \vec u)$ and increases with ${\hat{\sigma }}^{2}(t, \vec u)$, and the effect of ${\hat{\mu }}(t, \vec u)$ depends on ${\hat{\sigma }}^{2}(t, \vec u)$, and vice-versa. Alone, ${\hat{\mu }}(t, \vec u)$ and ${\hat{\sigma }}^{2}(t, \vec u)$ cause the tapir to double her home range (panels D and E), but together they result in an approximate 15-fold change in home-range size (observed range: 0.8 to 12.4 km^2^; see panel F). Additionally, note how high NDVI values (${\hat{\mu }}(t, \vec u) > 0.8$) cause ${\hat{\sigma }}^{2}(t, \vec u)$ to have little to no effect on home-range size, as indicated by the vertical contour line in panel F. Similar conclusions can be drawn for the animal’s diffusion (i.e., area covered per unit time), which is a more appropriate measure of space use when animals are not range resident [82].

**Fig. 6:**
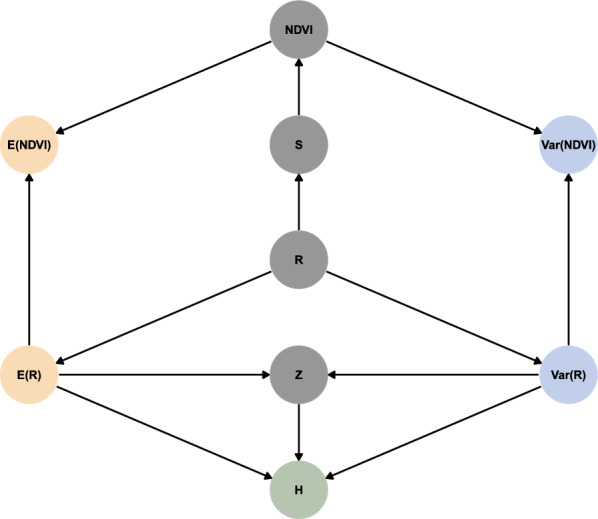
Directed acyclical graph assumed for inferring the causal effects of $\text {E}(R)$ and $\text {Var}(R)$ on *H*, where NDVI was used as a proxy for *R*. *Z* and *S* indicate confounds that result from habitat-level variables (e.g., competition, predation, etc.) and satellite-level variables (e.g., noise, cloud cover)

Quantifying the direct effects of $\text {E}(R)$ and $\text {Var}(R)$ on $H$ using empirical data is more complex than with simulated data, and it requires a different causal framework, particularly in the case of observational studies (as opposed to experimentally-controlled studies; see Fig. 6). Unlike with the simulations, $\text {E}(R)$ and $\text {Var}(R)$ are not controlled variables and instead depend on the distribution of $R$, which depends on a variety of other factors (that we exclude from the figure for simplicity). Both $\text {E}(R)$ and $\text {Var}(R)$ then impact $H$ as well as habitat-level variables (e.g., competition, predation, etc.; indicated as $Z$) that also affect $H$. Additionally, estimating $R$ via a proxy (NDVI) adds satellite-level noise and confounds [e.g., saturation, cloud cover, spatiotemporal averaging—indicated as $S$, see [83–85]. However, $\text {E}(R)$ and $\text {Var}(R)$ can be correlated to $\text {E}(\text {NDVI})$ and $\text {Var}(\text {NDVI})$, respectively, provided that analysts use models that are sufficiently smooth and flexible at the relevant spatiotemporal scale [86]. We discuss this in further detail in the section below on the strengths and limitations of the empirical approach.

## Discussion

The amount of space organisms use is determined by a multitude of factors [16], but the search for resources is often a main driver of how much and where organisms move. This paper builds on earlier theoretical work ([13], e.g., [18, 19]) and presents a unifying hypothesis that describes the effects of resource abundance and stochasticity on organisms’ range sizes. We use quantitative simulations and an empirical case study to support the hypothesis and show that it provides a simple framework for understanding how motile organisms adapt their movement in dynamic environments. Separately, resource abundance and stochasticity have simple but opposing effects on organisms’ range sizes: $H$ decreases with $\text {E}(R)$ and increases with $\text {Var}(R)$. Together, the degree to which $\text {E}(R)$ affects $H$ depends on $\text {Var}(R)$, and vice-versa, so organisms’ responses to resource dynamics can be complex. The simulated and empirical results suggest qualitatively similar marginal effects of $\text {E}(R)$ and $\text {Var}(R)$, but there are differences in the estimated interactive effects. In the simulated data, $\text {Var}(R)$ has little effect when $\text {E}(R)$ is low and a strong effect when $\text {E}(R)$ is high, while the opposite is true for the empirical data. This difference is due to two reasons. Firstly, the shape and symmetry of bounded distributions such as Gamma ($R > 0$) and Beta ($0< R < 1$) distributions depend on both $\text {E}(R)$ and $\text {Var}(R)$ (figs. A3, A4), but $\text {Var}(R)$ does not affect the shape of a Gamma distribution as much if $\text {E}(R)$ is low (fig. B3). Secondly, and perhaps more interestingly, the simulation approach does not account for real-world adaptations to $\text {E}(R)$ and $\text {Var}(R)$ such as selective space use, which are included (but not explicitly accounted for) in the empirical approach. Below we discuss the strengths and limitations of each approach.

### Strengths and limitations of the simulation-based approach

Our simulations are based on a simplistic environment with many assumptions that allowed us to estimate how resource abundance and stochasticity affect organisms’ home-range sizes if organisms can only respond to changes by adapting the amount of time spent searching for food (with no energetic cost to movement). The use of continuous-time movement models coupled with few drivers of movement supported realistic data that could be explained by straightforward causal models. The absence of confounding variables (e.g., predator avoidance, territoriality, competition, landscape connectivity; see Fig. 2) or sample size limitation allowed us to ensure estimates were accurate and robust (sensitivity analysis available in Appendix B).

Deviations from the simulations offer a means of detecting when the underlying assumptions are inappropriate and how additional factors may affect organisms’ responses to changes in $\text {E}(R)$ and $\text {Var}(R)$. For example, energetic costs of movement are often non-negligible and depend on organism size [40], movement speed [40], and ambient temperature [1, 87]. In addition, an organism may alter its movement behavior, physiology, and energetic needs to buffer itself against changes in $\text {E}(R)$ and $\text {Var}(R)$ by using space selectively [68, 88–90] and adapting their behavior and physiology over time [18, 69]. Before or during periods of scarcity, organisms may cache resources [91], build up fat reserves [45], enter states of dormancy [92–94], or even pause fetal growth [7]. However, organisms may be unable to respond to changes in $\text {E}(R)$ and $\text {Var}(R)$ optimally due to various reasons, including limited perceptive range [61], lack of experience [9, 47, 63–65, 95], avoidance of competitors and predators [14, 96], or a physiology that is not amenable to things like hibernation or fat storage. Thus, organisms may relocate their range to a sub-optimal location [33, 34, 97, 98], which may exacerbate the effects of $\text {E}(R)$ and $\text {Var}(R)$ on both mean range size and the variance around it.

### Strengths and limitations of the empirical approach

There are two main advantages of taking an empirical approach. Firstly, modeling real-world animal movement data can produce scale-appropriate and easily interpretable estimates. Secondly, empirical data contain information on the effects of $\text {E}(R)$, $\text {Var}(R)$, and confounding variables without having to design complex and time-consuming simulations. However, it is not always possible to quantify confounding variables. For example, while there may be some appropriate proxies of competition, such as density of competitors, these variables may be hard to quantify, and they may not account for the confounding effects appropriately (i.e., the presence of competitors may not reflect competitive pressure). This is problematic if one is interested in estimating the direct causal effect of $\text {E}(R)$ and $\text {Var}(R)$, which requires removing any non-negligible confounding effects [75].

Similarly, if $R$ non-measurable (as is often the case), $R$ must be estimated with proxies such as NDVI [76], which may introduce complexities. While $R$ and NDVI are correlated for many species (e.g., [45, 46, 95, 99–101]), the relationship between the two can be weak [84], satellite-dependent [85], and nonlinear [83, 85]. This complexity can introduce two sources of bias: ecosystem-level biases (indicated as $Z$ in the directed acyclical graph in Fig. 6) and satellite-level confounding variables ($S$ in Fig. 6). Examples of ecosystem-level biases are the effects of competition, predation, habitat connectivity, and movement costs, all of which can depend on habitat quality, and, consequently, be correlated nonlinearly to $R$ and NDVI [35, 102]. Resource-rich patches can attract larger amounts of competitors [14] and predators [20], which may, in turn, increase pressures from competition and predation [15, 39]. However, such pressures may result in both an expansion of the range [35, 102] or a contraction, since larger ranges can be harder to defend and result in higher movement costs [35, 103] and encounter rates [104]. Satellite-level confounds include information loss due to coarse spatiotemporal resolution [83, 85], satellite-level error [83, 85, 105], and other limitations of remote sensing (e.g., inability to quantify specific resources or small-scale resource depletion). However, nonlinear models such as Generalized Additive Models [106] can help account for preferences for intermediate values of remotely-sensed $R$ (e.g., young grass rather than mature grasslands, see [85]).

## Conclusions

The work presented here provides a unifying framework for viewing movement as a response to resource abundance and stochasticity. We provide a sensible and unifying hypothesis of the effects of $\text {E}(R)$ and $\text {Var}(R)$ on organisms’ range sizes and movement behavior. We demonstrate that organisms’ range sizes decrease with resource abundance, increase with resource stochasticity, and that the effects of $\text {Var}(R)$ can depend strongly on $\text {E}(R)$.

Recent advances in computational power have greatly increased analysts’ ability to fit computationally demanding models [107, 108] that allow biologists to move beyond only considering changes in mean conditions. By accounting for changes in stochasticity, we can start developing a more comprehensive understanding of how organisms adapt to the dynamic environments organisms live in, including recent changes in climate [109] and increases in the frequency and intensity of extreme events [66, 67, 110–112].

## Supplementary Information

Supplementary file 1.

Supplementary file 2.

Supplementary file 3.

## Data Availability

All code and data used for this manuscript is available on GitHub at https://github.com/QuantitativeEcologyLab/hr-resource-stoch, with the exception of two simulated datasets that were greater than 100 MB and the tapir data. The simulated data can be produced by running the scripts in the repository, while the tapir data is available at https://github.com/StefanoMezzini/tapirs.

## Abbreviations

*H*: Range size
${\hat{H}}_{95\%}$: Estimated $95\%$ home range size
*C*: Resource consumption rate
*R*: Resources
*t*: Moment in time
$\vec u$: Location in space (vector of coordinates)
$$\text {E}(R)$$: Resource abundance
$\mu (t)$: Resource abundance as a function of time
$\mu (t, \vec u)$: Resource abundance as a function of time and space
$$\text {Var}(R)$$: Resource stochasticity
$\sigma ^2(t)$: Resource stochasticity as a function of time
$\sigma ^2(t, \vec u)$: Resource stochasticity as a function of time and space
$\hat{\varsigma ^2}$: Estimated positional variance
$\text{Gamma} (\mu , \sigma ^2)$: Gamma distribution with mean $\mu$ and variance $\sigma ^2$
NDVI: Normalized Difference Vegetation Index

## References

[1] Hou R, Chapman CA, Jay O, Guo S, Li B, Raubenheimer D. Cold and hungry: combined effects of low temperature and resource scarcity on an edge-of-range temperate primate, the golden snub-nose monkey. Ecography. 2020;43:1672–82. 10.1111/ecog.05295.

[2] Le Bot T, Lescroël A, Fort J, Péron C, Gimenez O, Provost P, et al. Fishery discards do not compensate natural prey shortage in northern gannets from the English channel. Biol Cons. 2019;236:375–84.

[3] Dai Pra R, Mohr SM, Merriman DK, Bagriantsev SN, Gracheva EO. Ground squirrels initiate sexual maturation during hibernation. Current Biology. 2022;32:1822-1828.e4. 35245461 10.1016/j.cub.2022.02.032

[4] Rocha JL, Godinho R, Brito JC, Nielsen R. Life in deserts: the genetic basis of mammalian desert adaptation. Trends Ecol Evol. 2021;36:637–50. 33863602 10.1016/j.tree.2021.03.007 PMC12090818

[5] Wessling EG, Deschner T, Mundry R, Pruetz JD, Wittig RM, Kühl HS. Seasonal variation in physiology challenges the notion of chimpanzees (*Pan troglodytes* verus) as a forest-adapted species. Front Ecol Evol. 2018;6:60. 10.3389/fevo.2018.00060/full.

[6] Stefanescu C, Ubach A, Wiklund C. Timing of mating, reproductive status and resource availability in relation to migration in the painted lady butterfly. Anim Behav. 2021;172:145–53.

[7] Schmidt NM, Grøndahl C, Evans AL, Desforges J-P, Blake J, Hansen LH, et al. On the interplay between hypothermia and reproduction in a high arctic ungulate. Sci Rep. 2020;10:1514. 32001737 10.1038/s41598-020-58298-8 PMC6992616

[8] Douglas DJT, Pearce-Higgins JW. Relative importance of prey abundance and habitat structure as drivers of shorebird breeding success and abundance: drivers of shorebird breeding success and abundance. Anim Conserv. 2014;17:535–43. 10.1111/acv.12119.

[9] Foley C, Pettorelli N, Foley L. Severe drought and calf survival in elephants. Biol Lett. 2008;4:541–4. 10.1098/rsbl.2008.0370. 18682358 10.1098/rsbl.2008.0370 PMC2610102

[10] Berger J, Hartway C, Gruzdev A, Johnson M. Climate degradation and extreme icing events constrain life in cold-adapted mammals. Sci Rep. 2018;8:1156. 29348632 10.1038/s41598-018-19416-9 PMC5773676

[11] Van Haastert PJM, Bosgraaf L. Food searching strategy of amoeboid cells by starvation induced run length extension. PLoS ONE. 2009;4: e6814. 10.1371/journal.pone.0006814. 19714242 10.1371/journal.pone.0006814 PMC2729374

[12] Taub DR, Goldberg D. Root system topology of plants from habitats differing in soil resource availability. Funct Ecol. 1996;10:258.

[13] Harestad AS, Bunnel FL. Home range and body weight—a reevaluation. Ecology. 1979;60:389–402. 10.2307/1937667.

[14] Kacelnik A, Krebs JR, Bernstein C. The ideal free distribution and predator–prey populations. Trends Ecol Evol. 1992;7:50–5. 21235950 10.1016/0169-5347(92)90106-L

[15] Charnov EL. Optimal foraging, the marginal value theorem. Theor Popul Biol. 1976;9:129–36. 1273796 10.1016/0040-5809(76)90040-x

[16] Nathan R, Getz WM, Revilla E, Holyoak M, Kadmon R, Saltz D, et al. A movement ecology paradigm for unifying organismal movement research. Proc Natl Acad Sci USA. 2008;105:19052–9. 10.1073/pnas.0800375105. 19060196 10.1073/pnas.0800375105 PMC2614714

[17] Burt WH. Territoriality and home range concepts as applied to mammals. J Mammal. 1943;24:346. 10.2307/1374834.

[18] Southwood TRE. Habitat, the templet for ecological strategies? J Anim Ecol. 1977;46:336.

[19] Stephens DW, Charnov EL. Optimal foraging: Some simple stochastic models. Behav Ecol Sociobiol. 1982;10:251–63. 10.1007/BF00302814.

[20] Duncan C, Nilsen EB, Linnell JDC, Pettorelli N. Life-history attributes and resource dynamics determine intraspecific home-range sizes in Carnivora. Remote Sens Ecol Conserv. 2015;1:39–50. 10.1002/rse2.6.

[21] Rizzuto M, Leroux SJ, Vander Wal E, Richmond IC, Heckford TR, Balluffi-Fry J, et al. Forage stoichiometry predicts the home range size of a small terrestrial herbivore. Oecologia. 2021;197:327–38. 10.1007/s00442-021-04965-0. 34131817 10.1007/s00442-021-04965-0

[22] Broekman MJE, Hilbers JP, Hoeks S, Huijbregts MAJ, Schipper AM, Tucker MA. Environmental drivers of global variation in home range size of terrestrial and marine mammals. J Anim Ecol. 2024;93:488–500. 10.1111/1365-2656.14073. 38459628 10.1111/1365-2656.14073

[23] Singh NJ, Börger L, Dettki H, Bunnefeld N, Ericsson G. From migration to nomadism: Movement variability in a northern ungulate across its latitudinal range. Ecol Appl. 2012;22:2007–20. 10.1890/12-0245.1. 23210316 10.1890/12-0245.1

[24] Wheat RE, Lewis SB, Wang Y, Levi T, Wilmers CC. To migrate, stay put, or wander? Varied movement strategies in bald eagles (*Haliaeetus leucocephalus*). Mov Ecol. 2017;5:9. 10.1186/s40462-017-0102-4. 28484599 10.1186/s40462-017-0102-4 PMC5418703

[25] Teitelbaum CS, Mueller T. Beyond migration: causes and consequences of nomadic animal movements. Trends Ecol Evol. 2019;34:569–81. 30885413 10.1016/j.tree.2019.02.005

[26] Chevin L-M, Lande R, Mace GM. Adaptation, plasticity, and extinction in a changing environment: towards a predictive theory. PLoS Biol. 2010;8: e1000357. 10.1371/journal.pbio.1000357. 20463950 10.1371/journal.pbio.1000357 PMC2864732

[27] Herfindal I, Linnell JDC, Odden J, Nilsen EB, Andersen R. Prey density, environmental productivity and home-range size in the Eurasian lynx (*Lynx lynx*). J Zool. 2005;265:63–71. 10.1017/S0952836904006053.

[28] Nilsen EB, Herfindal I, Linnell JDC. Can intra-specific variation in carnivore home-range size be explained using remote-sensing estimates of environmental productivity? Écoscience. 2005;12:68–75. 10.2980/i1195-6860-12-1-68.1.

[29] Medici EP, Mezzini S, Fleming CH, Calabrese JM, Noonan MJ. Movement ecology of vulnerable lowland tapirs between areas of varying human disturbance. Mov Ecol. 2022;10:14. 10.1186/s40462-022-00313-w. 35287742 10.1186/s40462-022-00313-w PMC8919628

[30] Lindstedt SL, Boyce MS. Seasonality, fasting endurance, and body size in mammals. Am Nat. 1985;125:873–8. 10.1086/284385.

[31] Morellet N, Bonenfant C, Börger L, Ossi F, Cagnacci F, Heurich M, et al. Seasonality, weather and climate affect home range size in roe deer across a wide latitudinal gradient within Europe. J Anim Ecol. 2013;82:1326–39. 10.1111/1365-2656.12105. 23855883 10.1111/1365-2656.12105

[32] Fjelldal MA, Wright J, Stawski C. Nightly torpor use in response to weather conditions and individual state in an insectivorous bat. Oecologia. 2021;197:129–42. 10.1007/s00442-021-05022-6. 34455495 10.1007/s00442-021-05022-6 PMC8445878

[33] Tórrez-Herrera LL, Davis GH, Crofoot MC. Do monkeys avoid areas of home range overlap because they are dangerous? A test of the risk hypothesis in white-faced capuchin monkeys (*Cebus capucinus*). Int J Primatol. 2020;41:246–64. 10.1007/s10764-019-00110-0.

[34] Rich LN, Mitchell MS, Gude JA, Sime CA. Anthropogenic mortality, intraspecific competition, and prey availability influence territory sizes of wolves in montana. J Mammal. 2012;93:722–31. 10.1644/11-MAMM-A-079.2.

[35] Jetz W, Carbone C, Fulford J, Brown JH. The scaling of animal space use. Science. 2004;306:266–8. 10.1126/science.1102138. 15472074 10.1126/science.1102138

[36] Harvey PH, Clutton-Brock TH. Primate home-range size and metabolic needs. Behav Ecol Sociobiol. 1981;8:151–5. 10.1007/BF00300828.

[37] Baldwin R, Bywater A. Nutritional energetics of animals. Annu Rev Nutr. 1984;4:101–14. 10.1146/annurev.nu.04.070184.000533. 6087859 10.1146/annurev.nu.04.070184.000533

[38] Reich PB. Body size, geometry, longevity and metabolism: do plant leaves behave like animal bodies? Trends Ecol Evol. 2001;16:674–80.

[39] Brown JS, Laundre JW, Gurung M. The ecology of fear: optimal foraging, game theory, and trophic interactions. J Mammal. 1999;80:385–99. 10.2307/1383287.

[40] Taylor CR, Heglund NC, Maloiy GM. Energetics and mechanics of terrestrial locomotion. I. Metabolic energy consumption as a function of speed and body size in birds and mammals. J Exp Biol. 1982;97:1–21. 7086334 10.1242/jeb.97.1.1

[41] Relyea RA, Lawrence RK, Demarais S. Home range of desert mule deer: testing the body-size and habitat-productivity hypotheses. J Wildl Manag. 2000;64:146.

[42] Dawe KL, Bayne EM, Boutin S. Influence of climate and human land use on the distribution of white-tailed deer (*Odocoileus virginianus*) in the western boreal forest. Can J Zool. 2014;92:353–63. 10.1139/cjz-2013-0262.

[43] Berger-Tal O, Saltz D. Invisible barriers: anthropogenic impacts on inter- and intra-specific interactions as drivers of landscape-independent fragmentation. Philos Trans R Soc B. 2019;374:20180049. 10.1098/rstb.2018.0049. 10.1098/rstb.2018.0049 PMC6710564 31352896

[44] Samarra FIP, Tavares SB, Béesau J, Deecke VB, Fennell A, Miller PJO, et al. Movements and site fidelity of killer whales (*Orcinus orca*) relative to seasonal and long-term shifts in herring (*Clupea harengus*) distribution. Mar Biol. 2017;164:159. 10.1007/s00227-017-3187-9.

[45] Middleton AD, Merkle JA, McWhirter DE, Cook JG, Cook RC, White PJ, et al. Green-wave surfing increases fat gain in a migratory ungulate. Oikos. 2018;127:1060–8. 10.1111/oik.05227.

[46] Geremia C, Merkle JA, Eacker DR, Wallen RL, White PJ, Hebblewhite M, et al. Migrating bison engineer the green wave. Proc Natl Acad Sci USA. 2019;116:25707–13. 10.1073/pnas.1913783116. 31754040 10.1073/pnas.1913783116 PMC6925981

[47] Polansky L, Kilian W, Wittemyer G. Elucidating the significance of spatial memory on movement decisions by African savannah elephants using state-space models. Proc R Soc B. 2015;282:20143042. 10.1098/rspb.2014.3042. 25808888 10.1098/rspb.2014.3042 PMC4389615

[48] Nandintsetseg D, Bracis C, Leimgruber P, Kaczensky P, Buuveibaatar B, Lkhagvasuren B, et al. Variability in nomadism: environmental gradients modulate the movement behaviors of dryland ungulates. Ecosphere. 2019. 10.1002/ecs2.2924.

[49] Teitelbaum CS, Fagan WF, Fleming CH, Dressler G, Calabrese JM, Leimgruber P, et al. How far to go? Determinants of migration distance in land mammals. Ecol Lett. 2015;18:545–52. 10.1111/ele.12435. 25865946 10.1111/ele.12435

[50] Poessel SA, Woodbridge B, Smith BW, Murphy RK, Bedrosian BE, Bell DA, et al. Interpreting long-distance movements of non-migratory golden eagles: prospecting and nomadism? Ecosphere. 2022. 10.1002/ecs2.4072.

[51] Pretorius MD, Leeuwner L, Tate GJ, Botha A, Michael MD, Durgapersad K, et al. Movement patterns of lesser flamingos *Phoeniconaias minor*: nomadism or partial migration? Wildl Biol. 2020;2020:1–11. 10.2981/wlb.00728.

[52] Bista D, Baxter GS, Hudson NJ, Lama ST, Murray PJ. Effect of disturbances and habitat fragmentation on an arboreal habitat specialist mammal using GPS telemetry: a case of the red panda. Landsc Ecol. 2022;37:795–809. 10.1007/s10980-021-01357-w. 34720409 10.1007/s10980-021-01357-w PMC8542365

[53] Bradsworth N, White JG, Rendall AR, Carter N, Whisson DA, Cooke R. Using thresholds to determine priorities for apex predator conservation in an urban landscape. Landsc Urban Plan. 2022;228: 104559.

[54] McClintic LF, Taylor JD, Jones JC, Singleton RD, Wang G. Effects of spatiotemporal resource heterogeneity on home range size of American beaver. J Zool. 2014;293:134–41. 10.1111/jzo.12128.

[55] Lucherini M, Lovari S. Habitat richness affects home range size in the red fox *Vulpes vulpes*. Behav Proc. 1996;36:103–5. 10.1016/0376-6357(95)00018-6 24896422

[56] Watson J. Ferruginous hawk (*Buteo regalis*) home range and resource use on northern grasslands in Canada. 2020. 10.13140/RG.2.2.32404.32648

[57] Simcharoen A, Savini T, Gale GA, Simcharoen S, Duangchantrasiri S, Pakpien S, et al. Female tiger *Panthera tigris* home range size and prey abundance: important metrics for management. Oryx. 2014;48:370–7.

[58] Campillo F, Lobry C. Effect of population size in a predator–prey model. Ecol Model. 2012;246:1–10.

[59] Lee S-H. Effects of the probability of a predator catching prey on predator–prey system stability. J Asia Pac Entomol. 2011;14:159–62.

[60] Levin SA. The problem of pattern and scale in ecology: the Robert H. MacArthur award lecture. Ecology. 1992;73:1943–67. 10.2307/1941447.

[61] Steixner-Kumar S, Gläscher J. Strategies for navigating a dynamic world. Science. 2020;369:1056–7. 10.1126/science.abd7258. 32855326 10.1126/science.abd7258

[62] Mueller T, O’Hara RB, Converse SJ, Urbanek RP, Fagan WF. Social learning of migratory performance. Science. 2013;341:999–1002. 10.1126/science.1237139. 23990559 10.1126/science.1237139

[63] Abrahms B, Hazen EL, Aikens EO, Savoca MS, Goldbogen JA, Bograd SJ, et al. Memory and resource tracking drive blue whale migrations. Proc Natl Acad Sci USA. 2019;116:5582–7. 10.1073/pnas.1819031116. 30804188 10.1073/pnas.1819031116 PMC6431148

[64] Falcón-Cortés A, Boyer D, Merrill E, Frair JL, Morales JM. Hierarchical, memory-based movement models for translocated elk (*Cervus canadensis*). Front Ecol Evol. 2021;9: 702925. 10.3389/fevo.2021.702925/full.

[65] Fagan WF, Lewis MA, Auger-Méthé M, Avgar T, Benhamou S, Breed G, et al. Spatial memory and animal movement. Ecol Lett. 2013;16:1316–29. 10.1111/ele.12165. 23953128 10.1111/ele.12165

[66] Logares R, Nuñez M. Black swans in ecology and evolution: the importance of improbable but highly influential events. Ideas in Ecology and Evolution. 2012. https://ojs.library.queensu.ca/index.php/IEE/article/view/4311

[67] Anderson SC, Branch TA, Cooper AB, Dulvy NK. Black-swan events in animal populations. Proc Natl Acad Sci. 2017;114:3252–7. 10.1073/pnas.1611525114. 28270622 10.1073/pnas.1611525114 PMC5373335

[68] Riotte-Lambert L, Matthiopoulos J. Environmental predictability as a cause and consequence of animal movement. Trends Ecol Evol. 2020;35:163–74. 31699411 10.1016/j.tree.2019.09.009

[69] Levins RA. Evolution in changing environments: some theoretical explorations. 3. printing. Princeton: Princeton University Press; 1974.

[70] Van Baalen M, Křivan V, Van Rijn PCJ, Sabelis MW. Alternative food, switching predators, and the persistence of predator–prey systems. Am Nat. 2001;157:512–24. 10.1086/319933. 18707259 10.1086/319933

[71] Fleming CH, Calabrese JM. Ctmm: continuous-time movement modeling. 2021. https://github.com/ctmm-initiative/ctmm, https://groups.google.com/g/ctmm-user

[72] R Core Team. R: a language and environment for statistical computing. Vienna: R Foundation for Statistical Computing; 2023.

[73] Gurarie E, Fleming CH, Fagan WF, Laidre KL, Hernández-Pliego J, Ovaskainen O. Correlated velocity models as a fundamental unit of animal movement: synthesis and applications. Mov Ecol. 2017;5:13. 28496983 10.1186/s40462-017-0103-3 PMC5424322

[74] Fleming CH, Calabrese JM, Mueller T, Olson KA, Leimgruber P, Fagan WF. From fine-scale foraging to home ranges: a semivariance approach to identifying movement modes across spatiotemporal scales. Am Nat. 2014;183:E154-67. 10.1086/675504. 24739204 10.1086/675504

[75] McElreath R. Statistical rethinking: a Bayesian course with examples in R and Stan. Boca Raton: CRC Press/Taylor & Francis Group; 2016.

[76] Pettorelli N, Ryan S, Mueller T, Bunnefeld N, Jedrzejewska B, Lima M, et al. The normalized difference vegetation index (NDVI): unforeseen successes in animal ecology. Clim Res. 2011;46:15–27.

[77] Noonan MJ, Fleming CH, Akre TS, Drescher-Lehman J, Gurarie E, Harrison A-L, et al. Scale-insensitive estimation of speed and distance traveled from animal tracking data. Mov Ecol. 2019;7:35. 10.1186/s40462-019-0177-1. 31788314 10.1186/s40462-019-0177-1 PMC6858693

[78] Noonan MJ, Tucker MA, Fleming CH, Akre TS, Alberts SC, Ali AH, et al. A comprehensive analysis of autocorrelation and bias in home range estimation. Ecol Monogr. 2019;89: e01344. 10.1002/ecm.1344.

[79] Alston JM, Fleming CH, Kays R, Streicher JP, Downs CT, Ramesh T, et al. Mitigating pseudoreplication and bias in resource selection functions with autocorrelation-informed weighting. Methods Ecol Evol. 2022. 10.1111/2041-210X.14025.

[80] Silva I, Fleming CH, Noonan MJ, Alston J, Folta C, Fagan WF, et al. Autocorrelation-informed home range estimation: a review and practical guide. Methods Ecol Evol. 2022;13:534–44. 10.1111/2041-210X.13786.

[81] Wood SN, Pya N, Säfken B. Smoothing parameter and model selection for general smooth models. J Am Stat Assoc. 2016;111:1548–63. 10.1080/01621459.2016.1180986.

[82] Calabrese JM, Fleming CH, Gurarie E. Ctmm: An span style="font-variant:small-caps;"r/span package for analyzing animal relocation data as a continuous-time stochastic process. Methods Ecol Evol. 2016;7:1124–32. 10.1111/2041-210X.12559.

[83] Fan X, Liu Y. A global study of NDVI difference among moderate-resolution satellite sensors. ISPRS J Photogramm Remote Sens. 2016;121:177–91.

[84] Gautam H, Arulmalar E, Kulkarni MR, Vidya TNC. NDVI is not reliable as a surrogate of forage abundance for a large herbivore in tropical forest habitat. Biotropica. 2019;51:443–56. 10.1111/btp.12651.

[85] Huang S, Tang L, Hupy JP, Wang Y, Shao G. A commentary review on the use of normalized difference vegetation index (NDVI) in the era of popular remote sensing. J For Res. 2021;32:1–6. 10.1007/s11676-020-01155-1.

[86] Pease BS. Ecological scales of effect vary across space and time. Ecography. 2024;2024: e07163. 10.1111/ecog.07163.

[87] Brown JH, Gillooly JF, Allen AP, Savage VM, West GB. Toward a metabolic theory of ecology. Ecology. 2004;85:1771–89. 10.1890/03-9000.

[88] Johnson DH. The comparison of usage and availability measurements for evaluating resource preference. Ecology. 1980;61:65–71. 10.2307/1937156.

[89] Rickbeil GJM, Merkle JA, Anderson G, Atwood MP, Beckmann JP, Cole EK, et al. Plasticity in elk migration timing is a response to changing environmental conditions. Glob Change Biol. 2019;25:2368–81. 10.1111/gcb.14629. 10.1111/gcb.14629 30908766

[90] Ranc N, Cagnacci F, Moorcroft PR. Memory drives the formation of animal home ranges: evidence from a reintroduction. Ecol Lett. 2022;25:716–28. 10.1111/ele.13869. 35099847 10.1111/ele.13869

[91] Nespolo RF, Mejias C, Bozinovic F. Why bears hibernate? Redefining the scaling energetics of hibernation. Proc R Soc B. 2022;289:20220456. 10.1098/rspb.2022.0456. 35473385 10.1098/rspb.2022.0456 PMC9043729

[92] Goldberg AR, Conway CJ. Hibernation behavior of a federally threatened ground squirrel: climate change and habitat selection implications. J Mammal. 2021;102:574–87.

[93] Reher S, Ehlers J, Rabarison H, Dausmann KH. Short and hyperthermic torpor responses in the Malagasy bat *Macronycteris commersoni* reveal a broader hypometabolic scope in heterotherms. J Comp Physiol B. 2018;188:1015–27. 10.1007/s00360-018-1171-4. 30121696 10.1007/s00360-018-1171-4

[94] Mohr SM, Bagriantsev SN, Gracheva EO. Cellular, molecular, and physiological adaptations of hibernation: the solution to environmental challenges. Annu Rev Cell Dev Biol. 2020;36:315–38. 10.1146/annurev-cellbio-012820-095945. 32897760 10.1146/annurev-cellbio-012820-095945

[95] Merkle JA, Sawyer H, Monteith KL, Dwinnell SPH, Fralick GL, Kauffman MJ. Spatial memory shapes migration and its benefits: evidence from a large herbivore. Ecol Lett. 2019;22:1797–805. 10.1111/ele.13362. 31412429 10.1111/ele.13362

[96] Fretwell SD, Lucas HL. On territorial behavior and other factors influencing habitat distribution in birds: I. Theoretical development. Acta Biotheor. 1969;19:16–36. 10.1007/BF01601953.

[97] Ciuti S, Northrup JM, Muhly TB, Simi S, Musiani M, Pitt JA, et al. Effects of humans on behaviour of wildlife exceed those of natural predators in a landscape of fear. PLoS ONE. 2012;7: e50611. 10.1371/journal.pone.0050611. 23226330 10.1371/journal.pone.0050611 PMC3509092

[98] Burson A, Stomp M, Greenwell E, Grosse J, Huisman J. Competition for nutrients and light: testing advances in resource competition with a natural phytoplankton community. Ecology. 2018;99:1108–18. 10.1002/ecy.2187. 29453803 10.1002/ecy.2187

[99] Phillips LB, Hansen AJ, Flather CH. Evaluating the species energy relationship with the newest measures of ecosystem energy: NDVI versus MODIS primary production. Remote Sens Environ. 2008;112:4381–92.

[100] Seigle-Ferrand J, Atmeh K, Gaillard J-M, Ronget V, Morellet N, Garel M, et al. A systematic review of within-population variation in the size of home range across ungulates: what do we know after 50 years of telemetry studies? Front Ecol Evol. 2021;8: 555429. 10.3389/fevo.2020.555429/full.

[101] Merkle JA, Monteith KL, Aikens EO, Hayes MM, Hersey KR, Middleton AD, et al. Large herbivores surf waves of green-up during spring. Proc R Soc B. 2016;283:20160456. 10.1098/rspb.2016.0456. 27335416 10.1098/rspb.2016.0456 PMC4936031

[102] Prox L, Farine D. A framework for conceptualizing dimensions of social organization in mammals. Ecol Evol. 2020;10:791–807. 10.1002/ece3.5936. 32015844 10.1002/ece3.5936 PMC6988527

[103] Grant JWA. Whether or not to defend? The influence of resource distribution. Mar Behav Physiol. 1993;23:137–53. 10.1080/10236249309378862.

[104] Martinez-Garcia R, Fleming CH, Seppelt R, Fagan WF, Calabrese JM. How range residency and long-range perception change encounter rates. J Theor Biol. 2020;498: 110267. 32275984 10.1016/j.jtbi.2020.110267

[105] Tian F, Fensholt R, Verbesselt J, Grogan K, Horion S, Wang Y. Evaluating temporal consistency of long-term global NDVI datasets for trend analysis. Remote Sens Environ. 2015;163:326–40.

[106] Wood SN. Generalized additive models: an introduction with R. 2nd ed. Boca Raton: CRC Press/Taylor & Francis Group; 2017.

[107] Nathan R, Monk CT, Arlinghaus R, Adam T, Alós J, Assaf M, et al. Big-data approaches lead to an increased understanding of the ecology of animal movement. Science. 2022;375:eabg1780. 10.1126/science.abg1780. 35175823 10.1126/science.abg1780

[108] Wood SN, Li Z, Shaddick G, Augustin NH. Generalized additive models for gigadata: modeling the UK black smoke network daily data. J Am Stat Assoc. 2017;112:1199–210. 10.1080/01621459.2016.1195744.

[109] Intergovernmental Panel On Climate Change. Climate change 2021—the physical science basis: working group I contribution to the sixth assessment report of the intergovernmental panel on climate change. 1st ed. Cambridge University Press; 2023. https://www.cambridge.org/core/product/identifier/9781009157896/type/book

[110] Grant PR, Grant BR, Huey RB, Johnson MTJ, Knoll AH, Schmitt J. Evolution caused by extreme events. Philos Trans R Soc B. 2017;372:20160146. 10.1098/rstb.2016.0146. 10.1098/rstb.2016.0146 PMC5434096 28483875

[111] Rypkema D, Tuljapurkar S. Modeling extreme climatic events using the generalized extreme value (GEV) distribution. In: Handbook of statistics. New York: Elsevier; 2021. p. 39–71.

[112] Yao Q, Fan J, Meng J, Lucarini V, Jensen HJ, Christensen K, et al. Emergence of universal scaling in weather extreme events. 2022. arXiv:2209.02292

